# Molecular differences of adipose-derived mesenchymal stem cells between non-responders and responders in treatment of  transphincteric perianal fistulas

**DOI:** 10.1186/s13287-021-02644-8

**Published:** 2021-11-24

**Authors:** Michaela Tencerova, Lilli Lundby, Steen Buntzen, Stig Norderval, Helene Tarri Hougaard, Bodil Ginnerup Pedersen, Moustapha Kassem

**Affiliations:** 1 Molecular Endocrinology and Stem Cell Research Unit, Department of Endocrinology and Metabolism, Odense University Hospital and Institute of Clinical Research, University of Southern Denmark, Odense, Denmark; 2 Department of Surgery, Pelvic Floor Unit, Aarhus University Hospital, Århus, Denmark; 3 Department of Gastrointestinal Surgery, University Hospital of North Norway, Tromsoe, Norway; 4 Department of Clinical Medicine, UiT The Arctic University of Norway, Tromsö, Norway; 5 Department of Radiology, Aarhus University Hospital, Aarhus, Denmark; 6 Department of Cellular and Molecular Medicine, Faculty of Health Sciences, University of Copenhagen, Copenhagen, Denmark; 7 Molecular Physiology of Bone, Institute of Physiology of the Czech Academy of Sciences, Videnska 1083, 142 20 Prague 4, Czech Republic

**Keywords:** Autologous adipose tissue graft injection, Transsphincteric perianal fistula, Adipose-derived mesenchymal stem cells, Stem cell potency, Fistula healing

## Abstract

**Background:**

Injection of autologous adipose tissue (AT) has recently been demonstrated to be an effective and safe treatment for anal fistulas. AT mesenchymal stem cells (AT-MSCs) mediate the healing process, but the relationship between molecular characteristics of AT-MSCs of the injected AT and fistula healing has not been adequately studied. Thus we aimed to characterize the molecular and functional properties of AT-MSCs isolated from autologous AT injected as a treatment of cryptogenic high transsphincteric perianal fistulas and correlate these findings to the healing process.

**Methods:**

27 patients (age 45 ± 2 years) diagnosed with perianal fistula were enrolled in the study and treated with autologous AT injected around the anal fistula tract. AT-MSCs were isolated for cellular and molecular analyses. The fistula healing was evaluated by MRI scanning after 6 months of treatment. AT-MSC phenotype was compared between responders and non-responders with respect to fistula healing.

**Results:**

52% of all patients exhibited clinical healing of the fistulas as evaluated 6 months after last injection. Cultured AT-MSCs in the responder group had a lower short-term proliferation rate and higher osteoblast differentiation potential compared to non-responder AT-MSCs. On the other hand, adipocyte differentiation potential of AT-MSCs was higher in non-responder group*.* Interestingly, AT-MSCs of responders exhibited lower expression of inflammatory and senescence associated genes such as *IL1B, NFKB, CDKN2A, TPB3,TGFB1*.

**Conclusion:**

Our data suggest that cellular quality of the injected AT-MSCs including cell proliferation, differentiation capacity and secretion of proinflammatory molecules may provide a possible mechanism underlying fistula healing. Furthermore, these biomarkers may be useful to predict a positive fistula healing outcome.

*Trial registration*: NTC04834609, Registered 6 April 2021. https://clinicaltrials.gov/ct2/show/NCT04834609

**Supplementary Information:**

The online version contains supplementary material available at 10.1186/s13287-021-02644-8.

## Introduction

Perianal fistulas of crypto-glandular origin are common with an incidence of 10–23 cases/100,000/year [1]. Symptoms vary from mild discomfort such as minimal secretion and pruritus to acute perianal sepsis. Surgical interventions are frequent in order to classify the fistula, establish proper drainage and promote healing. This will often have a major impact on patient’s social activity and quality of life [2].

A number of sphincter-preserving procedures have emerged during the last decades. The advancement flap has been considered as the gold standard with a success rate around 80% [3]. Other techniques such as fibrin glue, collagen paste and fistula plug have success rates between 20 and 50%. More recent procedures such as Ligation of the Intersphincteric Tract (LIFT), Video-Assisted Anal Fistula Treatment (VAAFT) and Fistula Laser Closing device (FiLaC) have shown a wide range of success rates between 50 and 94% [4–6]. However, the results of the sphincter-preserving procedures vary widely between centers and comparison between studies has been difficult as the majority of studies are uncontrolled case series with variable endpoints and difference in the duration of follow up [7].

Local injection of adipose tissue-derived mesenchymal stem cells (AT-MSCs) has emerged as a novel approach with promising outcome in the treatment of both cryptoglandular fistulas and fistulizing Crohn’s disease [8, 9]. A variety of approaches have been reported including the use of cultured autologous stem cells [10] or in vitro expanded allogenic AT-MSCs that showed in a large phase 3 clinical trial, 50% in intervention compared to 30% healing rate of anal fistulas in patients with Crohn’s disease in the control group at 24 weeks follow up [11]. Other clinical studies reported a positive effect of using autologous AT-MSCs on a closure rate (around 50%) in the patients with cryptoglandular perianal fistulas in short and long term follow up period using different techniques and concentration of stem cells [9, 12]. However, these cell products require laboratory facilities to isolate and expand the cells in vitro and manufacturing the product is time consuming and very expensive.

Alternatively, injection of autologous adipose tissue (AT) graft, has been suggested as a simple and inexpensive surgical procedure compared to cellular therapies [13–16]. Injection of freshly harvested autologous AT without cellular extraction was first used in a pilot study in the treatment of recurrent anovaginal fistulas by de Weerd and colleagues [16] and a following study published by the same group demonstrated an encouraging success rate of 77% in a large cohort of the patients with anovaginal fistulas [17].

While the use of autologous AT in the treatment of perianal fistulas is being tested in an increasing number of clinical trials [13–17], the relationship between cellular composition of the injected products and the clinical outcome is not clear. A plausible mechanism underlying the positive healing effects might be attributed to immunomodulatory and anti-inflammatory effects of paracrine factors produced by AT-MSCs. In support of this notion, studies by Serena and colleagues [18, 19] reported impairment of immune properties of AT-MSCs in patients with Crohn´s disease and metabolic diseases such as obesity and diabetes compared to healthy controls. However, the relationship between the functional properties of the injected cells and the clinical outcome, has not been studied in a prospective clinical study.

In the present study, we investigated cellular and molecular characteristics of AT-MSCs obtained from autologous AT therapy in patients with high transphincteric perianal fistulas of crytoglandular origin, which were correlated with the outcome of the fistula treatment.

## Materials and methods

### Patients

In the present prospective clinical study we recruited 27 patients in Aarhus, Denmark (18 F/9 M, age 45 ± 2 (25–72 years) and body mass index (BMI) 28.8 ± 1.0 kg/m^2^) with high transsphincteric perianal fistulas of cryptoglandular origin, defined as a transsphincteric single tract fistula involving 50% or more of the external anal sphincter, supra-sphincteric and extra-sphincteric fistulas according to Parks classification [20].

A clinical assessment of the patient prior to inclusion was undertaken by one of the responsible colorectal surgeons. If a seton was not in place at the time of assessment, the fistula was visualised with a probe, revised if necessary and a loose seton placed for at least 6 weeks prior to fat injection. Inclusion and exclusion criteria are listed in Table 1. An MRI of the pelvis was performed before inclusion and a standardised assessment was made by BG. Fistulas with secondary tracts and/or cavities were excluded. To identify a high transsphincteric fistula the length of the external sphincter distal to the level where the fistula tract transverses the external anal sphincter was determined and related in percent to the whole length of the external anal sphincter, including the puborectalis muscle for lateral and posterior fistulas.

**Table 1:** Definition of inclusion and exclusion criteria

	Definition
Inclusion criteria	High trans-sphincteric (> 50% of external sphincter), supra-sphincteric or extra-sphincteric fistulas The fistula confirmed and classified by an MRI Seton (> 6 weeks) prior to fat injection Informed, written consent
Exclusion criteria	Anovaginal fistula Active sepsis IBD, immunodeficiency, prior pelvic irradiation and fistula caused by malignancy Insulin dependent diabetes More than 4 prior attempts of closure with sphincter saving procedures Tobacco smoking or nicotine substitution of any kind 8 weeks prior to fat injection Pregnancy Psychiatric disorders BMI ≥ 35 or BMI < 20 kg/m^2^ Active tuberculosis Patient less than 18 years Unable to undergo MRI

### Functional outcome assessment of fistula

Functional anorectal disturbances and symptoms were recorded at baseline and 6 months after injection of autologous AT. Severity of faecal incontinence was evaluated by the St Mark’s Score [21]. Symptoms of obstructed defecation were assessed using the ODS-score [22]. If a patient had a diverting stoma, these data were not applicable. Urinary symptoms were registered using the ICIQ-UI-SF [23]. Number and kind of previous fistula surgery was recorded.

### Surgical procedure

The operations were performed by three colorectal surgeons (LL, SB, HH). The surgical procedure has been described previously [17, 24]. In brief, liposuction and injection of autologous AT were performed in one surgical procedure. The liquid fraction of the freshly harvested fat was separated by centrifugation and expelled. The fistula tract was curetted and the internal opening of the fistula was closed with sutures. The prepared AT was carefully injected around and into the fistula tract and the external opening left open for drainage. A part of of prepared AT (cca 20–30 ml) was used for adipose derived mesenchymal stem cell (AT-MSC) isolation for further molecular and cellular analysis at Stem Cell Research Unit, Odense University Hospital, Denmark in order to evaluate stem cell quality.

### Isolation of the adipose derived mesenchymal stem cells (AT-MSCs) obtained from liposuction

Aspirated AT from all 27 patients with transsphincteric fistulas was analyzed for stem cell characteristics. In brief, AT-MSCs were isolated according to the protocol of Yu and Gimble et al. with a few modifications [25]. AT was washed two times in PBS followed by collagenase digestion (*Invitrogen Col type I cat.n. 17,018–029,* c = 0.1% in 1% Bovine Serum Albumin (BSA) and Phosphate Buffered Saline (PBS) with Ca, Mg^++^) for 45 min-1 h at 37 °C in a shaking water bath, followed by centrifugation, red blood cells (RBC) lysis, filtration through 70 μm mesh and washing with PBS. Initially, the cells were plated in culture dishes in DMEM/F12 culture medium with 10% fetal bovine serum (FBS), incubated at 5% CO_2_ at 37 °C and then nourished by completely changing the medium once every 3 days along with a passage at 80% confluence. After expansion in vitro*,* AT-MSCs were sub-cultured and further studied in differentiation conditions to induce adipogenesis and osteogenesis.

### Functional assessment of AT-MSC cellular and molecular characteristics

#### Flow cytometry of AT-MSCs

Cell concentration was counted using a cell chamber. For the quantification of cell types among the isolated cells, the cells were immunophenotyped using a panel of fluorescence-conjugated antibodies. The cells were incubated with Fc- receptor blocking solution (Miltenyi) to block unspecific binding of antibodies, followed by incubation with specific antibodies according to manufacturer recommendations. Panel of used fluorescence-conjugated antibodies in the study as followed: PE-conjugated-anti-CD44 (Beckman Coulter, cat.no: A32537), PE-conjugated-anti-CD90 (Beckman Coulter, cat.no: IM3600U), PE-conjugated-anti-CD105 (Beckman Coulter, cat.no: A07414), FITC-conjugated-anti-CD271 (Biolegend, cat.no: 345104), FITC-conjugated-anti-CD31 (BD Pharmingen, cat.n. 555445), PE-conjugated-anti-CD34 (BD Pharmingen, cat.no: 555822), PE-conjugated-anti CD14 (BD Pharmingen, cat.no: 555398) and APC-conjugated-anti-CD45 (BD Pharmingen, cat.n. 555485), FITC-LEPR (R&D systems, cat.n. FAB867F), PE-SOX2 (R&D systems, cat.n. IC2018P), PE-CD49a (BD Pharmingen, cat.n. 559596). Following incubation for 30 min in the dark at 4 °C, cells were washed and analysed by BD LSR II (BD Biosciences). The flow cytometry analysis was performed by Kaluza 1.1 analysis software. Flow cytometric gating was defined based on relevant isotype controls.

#### Cell proliferation of AT-MSCs

Primary AT-MSCs were plated in 6-well plates at a density of 10,000 cells/well in a standard growth medium supplemented with 10% fetal bovine serum (FBS). Cells number was evaluated every day from 1 to 14 days. Cells were washed with phosphate buffered saline, harvested by trypsinization and manually counted in triplicate using Burker-Turk counting chambers (Thermo Fisher) [26].

### In vitro differentiation of AT-MSCs

#### Adipocyte differentiation and Oil Red O staining

Cells were plated at a density of 30.000 cells/cm^2^. Adipocytic induction media DMEM containing 10% FBS, 10% Horse serum (Gibco), 100 U/mL penicillin (Gibco), 100 μg/mL streptomycin (Gibco), 100 nM dexamethasone (Sigma-Aldrich), 0.25 mM 3-isobutyl-1-methyxanthine (IBMX), 1 μM BRL (Sigma-Aldrich), 3 μg/mL Insulin (Sigma-Aldrich) was changed every other day for 10 days.

At day 10 of adipocyte differentiation, cells were fixed in 4% paraformaldehyde for 10 min at room temperature then stained with Oil Red O (Sigma-Aldrich) to visualize the lipid content. Briefly, cells were rinsed in 3% isopropanol solution and stained with filtered Oil Red O solution (0.5 g in 100% isopropanol) for 1 h at room temperature (RT) [26].

#### Osteoblast differentiation and Alizarin red staining

The cells were plated at a density of 20.000 cells/cm^2^ in alpha MEM medium (Gibco) containing 10%

FBS, 100 U/mL penicillin (Gibco), 100 μg/mL streptomycin (Gibco). Osteoblast induction media composed of base medium supplemented with 10 mM B-glycerophosphate (Sigma-Aldrich), 10 nM dexamethasone (Sigma-Aldrich), 50 μg/mL Vitamin C (Sigma-Aldrich) and 50 μg/mL Vitamin D (Sigma-Aldrich) was replaced 1 day after the seeding. The medium was changed every other day for 10 days.

#### Alizarin red staining

Mineralized matrix formation at day 10 of osteoblast differentiation, was measured using Alizarin red staining [27]. Cells were fixed with 70% ice-cold ethanol for 1 h at − 20 °C before addition of AR-S (40 mM; Sigma-Aldrich) dissolved in distilled water (pH 4.2). The cells were stained for 10 min at RT. The level of calcium deposition was quantified by elution of AR-S following incubation in 10% cetylpyridinium chloride (Sigma-Aldrich) for 1 h at RT. The absorbance of the eluted dye was assessed at 570 nm in a FLU Ostar® Omega plate reader.

### Alkaline phosphatase (ALP) activity assay

Cells were incubated with naphthol AS-TR phosphate solution containing Fast Red TR (Sigma-Aldrich) as described previously [27]. Alkaline phosphatase activity was measured using p-nitrophenyl phosphate (Fluka Chemie) as substrate [28].

### RNA extraction and real time qRT-PCR

RNA was extracted using TRIzol then the Qiagen Rneasy Mini Kit (Qiagen) and reverse-transcribed using a RevertAid H Minus First Strand cDNA Synthesis Kit (Thermo Scientific). Quantitative real-time PCR was performed with an Applied Biosystems 7500 Real-Time PCR System using Fast SYBR Green Master Mix (Applied Biosystems) with specific primers (Additional file 1: Table S1). β-actin was used as an endogenous control. Results are expressed as delta-delta Ct values.

### Evaluation of treatment effect

All patients were clinically evaluated 6 weeks, 12 weeks and 6 months after the primary operation. A fistula was considered completely clinically healed if the patient had no symptom of discharge, no visible external opening in the perianal area and closed internal opening evaluated by digital rectal examination. If the fistula was not completely clinically healed at 6 weeks, a second operation was performed using the same technique as described and the follow up program was repeated.

MRI was performed together with completion of all questionnaires if the examination at 6 months after last injection demonstrated clinical healing. MRI definition of complete clinical healing was no visible fluid conducting tract or collection at former fistula site. Complications were registered according to Clavien-Dindo classification [29].

### Statistical analyses and ethical consideration

The statistical significance of the differences in the means of experimental groups (responders vs non-responders) were determined by unpaired t-test using GraphPad Prism 5.0a software. Data are presented as means ± SEM. *p* value < 0.05 was considered significant. All the statistical details of experiments can be found in the figure legends.

The study was performed according to the Declaration of Helsinki and was approved by the Regional Committee on Health Research Ethics in Aarhus, Denmark (M-2014-398-14). All participants signed informed consent prior to participation in the study. This study is registered as ClinicalTrials.gov (NTC04834609). All authors had access to the study data and reviewed and approved the final manuscript.

## Results

### Study population

This prospective longitudinal study was conducted from January 2015 to October 2017. Twenty-seven consecutive patients (18F/9M) with high transsphincteric anal fistulas of cryptoglandular origin were recruited at Aarhus University Hospital and treated with freshly harvested autologous AT graft injections. The patients ranged in age from 25 to 72 years at the time of operation, with a mean age of 45 ± 2 years and BMI 28.8 ± 1.0 kg/m^2^ (Table 2).

**Table 2:** Basic characteristics of the patients

	Whole cohort (*n* = 27)	Responders (*n* = 12)	Non-responders (*n* = 15)
Gender ratio (F/M)	18F/9M	9F/3M	9F/6M
Age (years)	45 ± 2	44 ± 3	46 ± 4
BMI (kg/m^2^)	28.8 ± 1.0	30.3 ± 1.6	27.6 ± 1.4

All fistulas were classified as high single tract transsphincteric involving more than 50% of the external anal sphincter. Five patients had undergone one prior operation, three patients had two prior operations and one patient had three prior attempts of fistula closure. The most preferred procedures were anal fistula plug, LIFT and advancement flap. Two patients had a covering stoma before the decision to undergo autologous AT graft injection for fistula treatment.

A total of 44 fat injection procedures were performed. Ten patients had one injection and seventeen patients had two injections. The mean volume of injected fat was 70 ml (47–97 ml) for the first injection and 69 ml (40–105 ml) for the second injection. Mean operation time was 85 min (57–148 min).

### Outcome of fistula healing

**Fig. 1:**
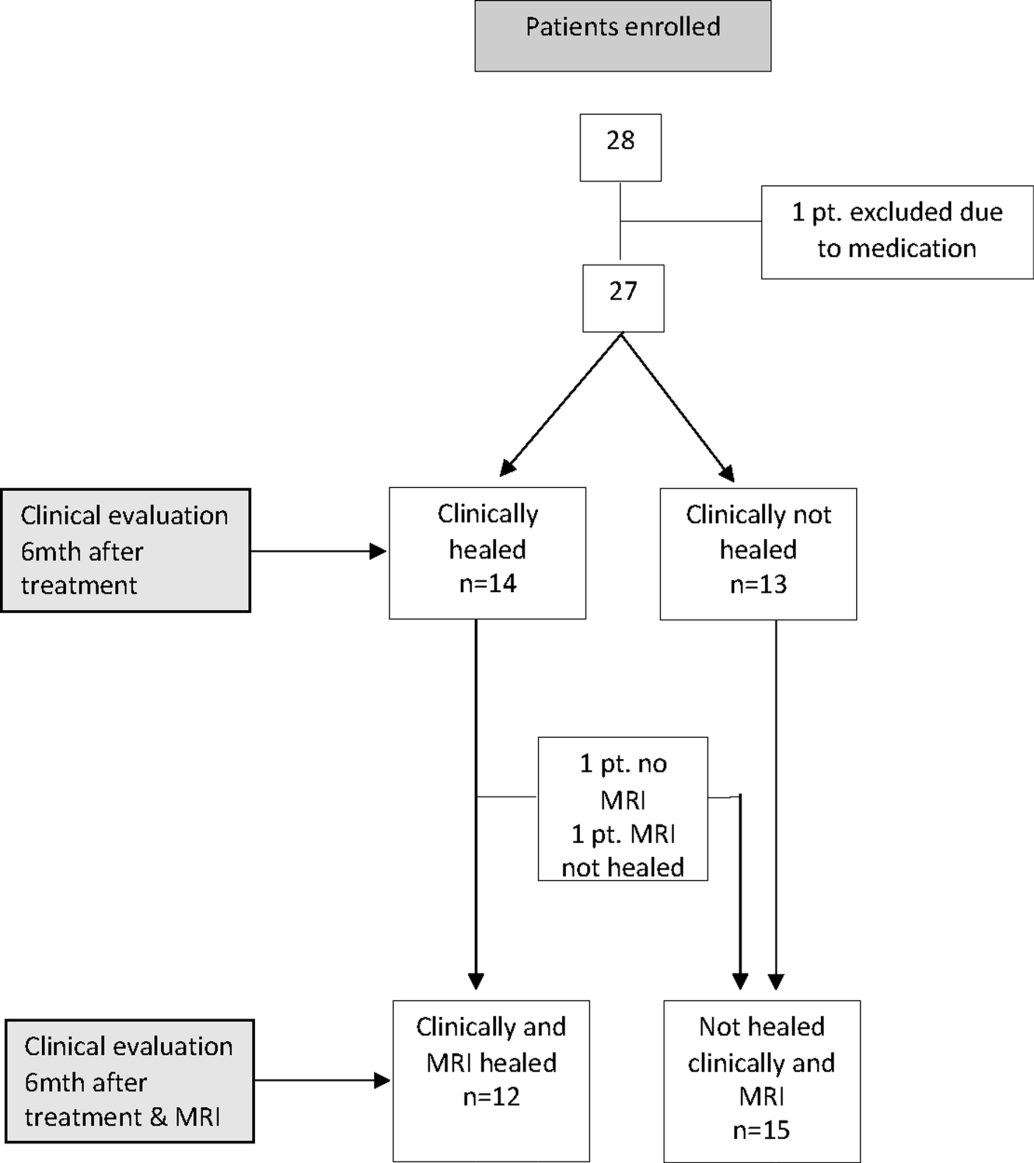
The flow chart of the enrollment of the patients and outcome of the study 6 months after last AT injection

Forteen patients (52%) had complete fistula healing on clinical examination 6 months after last injection with freshly collected autologous AT. Among these patients pelvic MRI confirmed complete fistula healing in twelve patients (44%) who were considered as *responders,* while fifteen patients exhibited persistent fistula and were considered as *non-responders*. One patient with a clinically healed fistula had a remaining gracile fistula tract, which was clearly reduced in size and fluid content compared with baseline pelvic MRI. Eighteen months later there was no clinical sign of recurrence of the fistula. Another patient with clinically healed fistula did not consent for the MRI at 6 months follow up. Two patients, both with a covering stoma, developed an abscess after 3 and 3 ½ months respectively. These data are depicted in a flow chart in Fig. 1. As shown in Table 2 the basic clinical characteristics of the responders and non-responders did not reveal any significant differences in age or BMI between the groups suggesting that the quality of AT product plays an important role in mediating the healing process.

### Fistula healing after single and repeated injections with adipose tissue

Ten patients healed clinically at 6 months follow up after one injection of AT. Seventeen patients had the procedure repeated and three of these patients healed 6 months after the second injection.

### Functional outcome of fistula treatment

Functional outcome scores were performed before the operation and at 6 months postoperatively. There was no difference in bowel function before and after operation. Mean St Mark’s score before the operation was 5 compared to 4.6 postoperatively (*p* = 0.47). The Altomare Obstructed defecation score was 3 before the operation and 4.1 after the operation (*p* = 0.52). None of the patients reported significant urinary problems neither before nor after the procedures.

### Clinical complications

Two patients (7.5%) developed an abscess requiring a surgical intervention (Clavien-Dindo IIIb) in the postoperative period. Proctalgia, minor discomfort and pain after liposuction procedure was experienced by some patients but the symptoms were resolved with paracetamol and ibuprofen (Clavien-Dindo I). Subcutaneous infections after lipoaspiration were not observed.

### Cellular and molecular characteristics of AT grafts

As there were no differences in clinical characteristics of responders and non-responders, we examined the hypothesis whether the quality of AT product contributed to the success of fistula healing process. In order to characterize the biological properties of injected AT, we investigated cellular and molecular characteristics of AT-MSCs isolated from AT (*n* = 27) at the beginning of the treatment and compared these parameters between the groups of responders and non-responders.

**Fig. 2:**
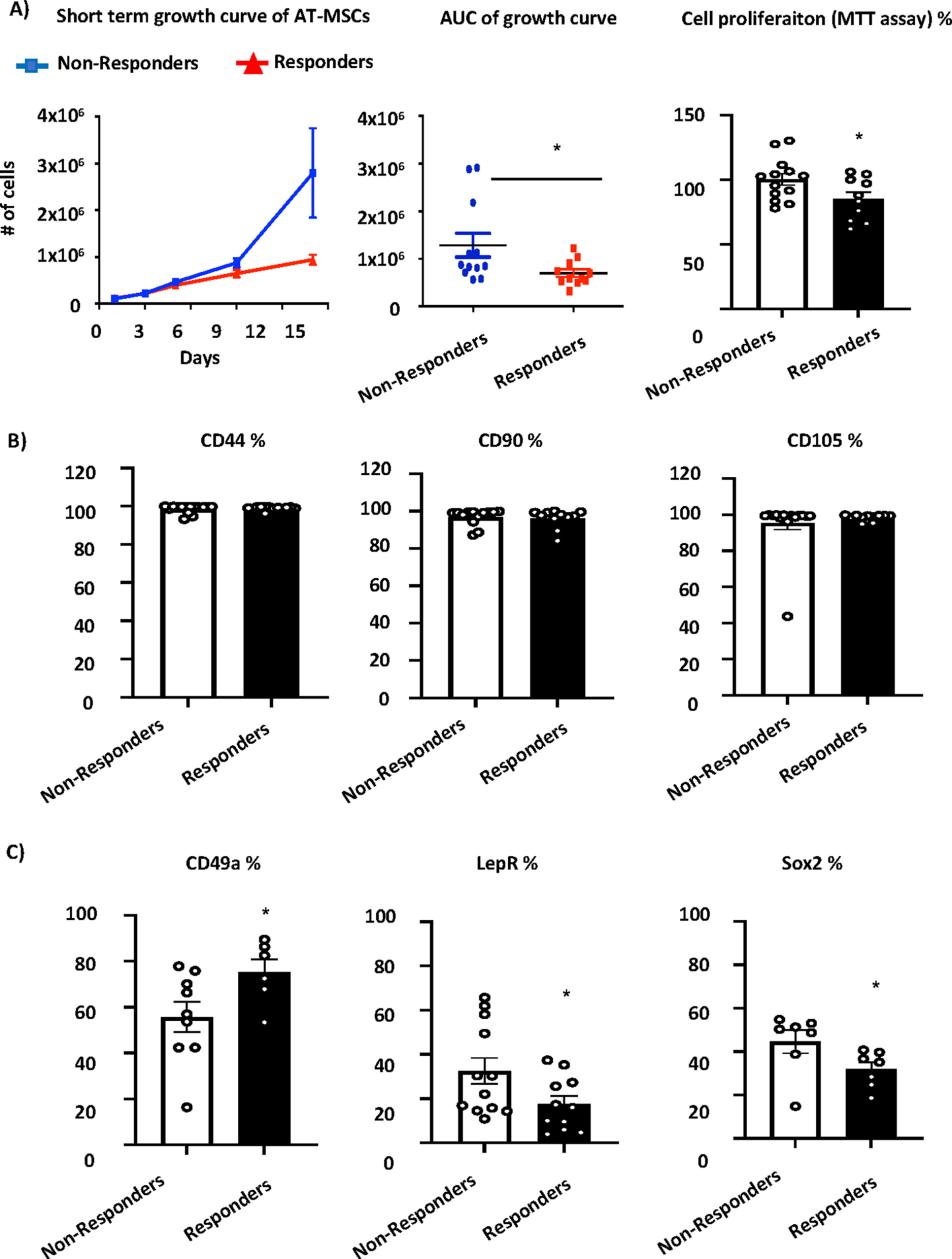
Cellular characteristics of AT-MSCs from responders and non-reponders in fistula treatment. AT-MSCs were established from non-responders (*n* = 15) and responders (*n* = 12) in AT graft fistula treatment. The cells were examined in undifferentiated state in passage 1. **A** Short-term proliferative rate, area under the curve (AUC) and cell proliferation rate measured by MTT assay (from left to right) of AT-MSCs in non-responder and responder group (*n* = 12–15). *p < 0.05, non-responders versus responders. Screening of stem cell surface marker expression, such as **B** CD44, CD90, CD105 and **C** CD49a, LEPR, and SOX2 measured using flow cytometry in AT-MSCs isolated from non-responder and responder subjects (*n* = 12–15). Data are presented as means ± SEM; *p < 0.05, non-responders versus responders, (two-tailed unpaired Student’s *t* test)

We detected no difference in the yield of isolated cells (0.13 × 10^6^ ± 0.01 × 10^6^ vs 0.12 × 10^6^ ± 0.02 × 10^6^, responders vs non-responders). AT-MSCs in the responder group had a lower short-term proliferation rate compared to non-responder group (Fig. 2A). Screening of stem cell surface markers by flow cytometry did not show any changes in expression profile of markers known to be expressed by AT-MSCs such as CD44, CD90, CD105 or CD271 (Fig. 2B). However, AT-MSCs in responder group had a higher expression of CD49a and lower expression of Leptin receptor (LEPR) and SOX2 (Fig. 2C). These markers were previously reported to be associated with multipotency and immuno-modulatory properties of AT-MSCs [30–32].

**Fig. 3:**
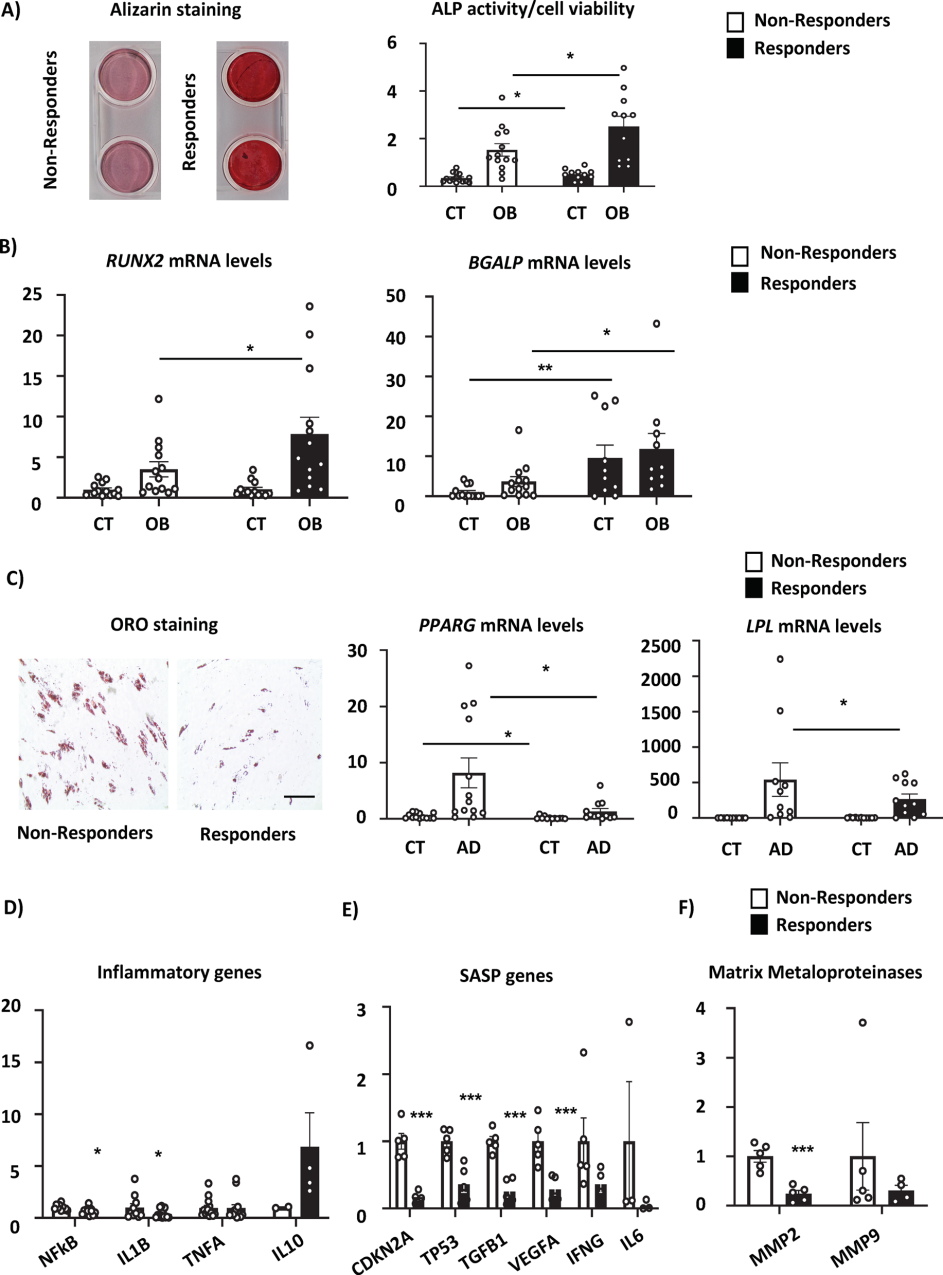
Differentiation potential and inflammatory profile of AT-MSCs from responders and non-responders in fistula treatment. AT-MSCs were established from non-responders (*n* = 15) and responders (*n* = 12) in AT graft fistula treatment. The cells were examined in undifferentiated and differentiated state in passage 2. Osteoblast differentiation potential of AT-MSCs evaluated by **A** Alizarin S staining and using quantification of alkaline phosphatase (ALP) activity represented as fold change (F.C.) over non-induced cells (day 7); **B** and gene expression of *RUNX2* and *BGALP* mRNA levels (*n* = 12–15); *p < 0.05: non-responders versus responders (two-tailed unpaired Student’s *t* test). Adipocyte differentiation potential of AT-MSCs evaluated by **C** Oil red O staining of mature adipocytes (magnification 10x, scale bar 100 μm) and gene expression of *PPARG* and *LPL* (n = 12–15); **D** Gene expression profile of pro-inflammatory (*NFKB, IL1B* and *TNFA*) and anti-inflammatory genes (*IL10*) in non-responder and responder AT-MSCs (*n* = 12–15). **E** Gene expression profile of senescence associated secretory phenotype (SASP) (*CDKN2A, TPB3, TGFB1, VEGFA, IFNG, IL6*) in non-responder and responder AT-MSCs (*n* = 5); **F** Gene expression profile of matrix metalloproteinases (*MMP2, MMP9*) in non-responder and responder AT-MSCs (*n* = 5). Data are presented as means ± SEM; *p < 0.05, **p < 0.01, ***p < 0.001: non-responders versus responders (two-tailed unpaired Student’s *t* test)

We also analyzed differentiation potential of AT-MSCs. The responder AT-MSCs exhibited higher osteoblast (OB) differentiation potential compared to non-responder AT-MSCs as measured by Alizarin S staining and alkaline phosphatase (ALP) activity (Fig. 3A) as well as gene expression of osteoblastic markers including *RUNX2* and *BGALP* (Fig. 3B). On the other hand, adipocyte (AD) differentiation potential of AT-MSCs was higher in non-responder group evaluated by Oil Red O staining and gene expression of *PPARG* and *LPL* (Fig. 3C). Importantly, AT-MSCs of responders exhibited lower gene expression of inflammatory genes such as *IL1B, NFKB* and a trend of increase of anti-inflammatory gene *IL10,* which was associated with a better fistula healing (Fig. 3D). Further, gene expression profiling of senescence associated secretory phenotype (SASP) genes (e.g. *CDKN2N, TP53, TGFB1, VEGFA*) (Fig. 3E) and matrix metalloproteinases related to tissue remodelling (*MMP2, MMP9*) (Fig. 3F) were increased in non-responder group in comparison to responder group.

## Discussion

In the present study we have demonstrated that injection of freshly harvested autologous AT obtained by liposuction, is a possible new approach for treatment of perianal fistula with a good healing rate of 44–52% at 6 months. We found a molecular phenotype of AT-MSCs isolated from AT graft characterized by several cellular characteristics associated with better healing of fistulas, which might be helpful in elucidating the mode of action and in predicting the effectiveness of AT graft therapy for perianal fistulas.

The observed healing rate of 44% in our patient population may be lower than reported by other investigators using AT engraftment in Fistula patients. The healing rate reported in a study by Naldini et al. [14] was 73%. This discrepancy may be related to patients characteristics including different age, metabolic comorbidities, fistula complications, defining of fistula healing or the features of cellular product. We employed centrifugation procedure followed by removal of the liquid fraction from the AT product, while Naldini et al. [14] employed a commercial processing kit (Lipogems®) to intraoperatively provide micro-fragmentation of AT for implantation. In addition, we based fistula healing on a very conservative criteria that included a combination of clinical examination and MRI imaging with no sign of fluid conducting tracts or sinuses, while Naldini et al. defined fistula healing as no discharge and closure of the internal and external opening.

We observed that clinical characteristics of the patients such as BMI, age or gender did not differ between the responder and non-responder group with respect to fistula healing, also the presence of any other clinical complications besides fistula, suggesting that characteristics of the cellular product plays an important role in determining the clinical outcome. In addition, responder group underwent one autolougous AT engraftment for successful fistula healing, while non-responder group did not heal even after second AT injection. This observation pointed out the fact that the cellular quality of AT is critical for a successful healing process in fistula treatment. Indeed, a number of differences of cellular characteristics of cultured AT-MSCs between responders versus non-responders have been highlighted. AT-MSCs of non-responder group exhibited higher proliferation rate compared to responders, which was accompanied with a higher inflammatory profile. These findings confirm the previous data of Zubkova et al. [33] reporting higher proliferation of AT-MSCs in presence of inflammatory environment of the cells. It is possible that this phenotype of the cells was induced due to their presence in an inflammatory microenvironment [18, 19]. Also gene expression profile of senescence associated genes along with genes related to extracellular matrix comfirmed more defective cellular properties of AT-MSCs in non-responder group compared to responders. In addition, screening of stromal stem cell surface markers of AT-MSCs by flow cytometry revealed an increase of CD49a, a member of integrins, which binds to extracellular matrix components as a part of the α1β1 integrin complex suggesting a presence of cells with higher regenerative properties [34]. In support of this notion, we observed that differentiation capacity of AT-MSCs in responder group shifted towards higher differentiation potency to OB than AD. OB differentiation is an in vitro surrogate marker for cellular ability for matrix production, which is important for tissue regeneration [35, 36]. On the other hand, AT-MSCs of non-responders exhibited higher capacity for AD differentiation and higher levels of proinflammatory cytokines and senescent markers. This cellular and molecular signature is reminiscent of what we have previously reported in bone marrow stromal stem cells associated with a high degree of AD differentiation that expressed higher production of reactive oxygen species, higher LEPR expression and pro-inflammatrory secretory phenotype [26]. Thus, our data corroborate that the cell composition of AT used in the fistula treatment and their secretory products are major contributors to the healing process and contribute to the success rate of the autologous AT injection treatment.

The present study has a unique strength. First, in contrast to previous studies, the clinical model employed was standarized as all patients exhibited a single track high sphincteric fistulas that were verified both clinically and with MRI imaging. Second, we employed strict criteria to determine healing of the fistulas following therapy. Third, we employed state-of-the-art characteristics of AT-MSCs suitable for clinical investigation.

On the other hand, our study has some limitations. We included only 27 patients. Moreover, the observed prognostic criteria have not been verified in an independent prospective cohort, which will need further follow up study to confirm these findings. Furthermore, as the study group was healthy, we did not characterize the patients biochemically or metabolically prior to the treatment. Assessment including lipid profile, fasting glucose/insulin levels and circulating levels of cytokines and adipokines would have provided insight into the in vivo microenvironment with respect to cellular characteristics [26].

## Conclusion

Using autologous fat graft injection in fistula treatment showed very promising and safe results in fistula healing. In this prospective study we identified a unique signature of AT-MSCs associated with better healing of fistulas. Lower proliferation rate, higher osteoblast differentiation capacity and lower secretion of proinflammatory molecules of AT-MSCs define the better cellular quality in responders compared to non-responders in the fistula treatment. These cellular characteristics were associated with better healing process in fistula patients. Our data provide insight into the importance of cellular quality of AT in determining the healing outcome of fistulas. Finally, our study provides some plausible mechanisms for the positive effects of AT graft therapy in fistula treatment.

## Supplementary Information

**Additional file 1:** List of primer sequences used for qRT-PCR.

## Data Availability

The data that support the findings of this study are available on request from the corresponding author.

## Abbreviations

AT: Adipose tissue
AT-MSCs: Adipose tissue-derived mesenchymal stem cells
AD: Adipocyte
ALP: Alkaline phosphatase
BMI: Body mass index
BSA: Bovine Serum Albumin
FBS: Fetal bovine serum
FiLaC: Fistula Laser Closing device
LEPR: Leptin receptor
LIFT: Ligation of the Intersphincteric Tract
MRI: Magnetic resonance imaging
OB: Osteoblast
PBS: Phosphate Buffered Saline
SASP: Senescence associated secretory phenotype
VAAFT: Video-Assisted Anal Fistula Treatment
