# Supplementary material for: Molecular differences of adipose-derived mesenchymal stem cells between non-responders and responders in treatment of transphincteric perianal fistulas
Source: Stem Cell Res Ther. 2021 Nov 24;12:586. doi: 10.1186/s13287-021-02644-8 (PMC8611942; doi:10.1186/s13287-021-02644-8)
Supplement: Supplementary file 1 — Additional file 1: List of primer sequences used for qRT-PCR. [file 13287_2021_2644_MOESM1_ESM.docx]

**Table S1**. List of primer sequences used for qRT-PCR.

| **Gene** | **Forward sequence** | **Reverse sequence** |
| --- | --- | --- |
| ***β-ACTIN*** | ATTGGCAATGAGCGGTTCCG | AGGGCAGTGATCTCCTTCTG |
| ***PPARG*** | CTCCTATTGACCCAGAAAGCGA | TGCCATGAGGGAGTTGGAAG |
| ***CEBPA*** | AAC CTT GTG CCT TGG AAA TG | CTG TAG CCT CGG GAA GGA G |
| ***FABP4*** | TGGGGGTGTCCTGGTACATGTGCAGAAAT | ACGCCTTTCATGACGCATTCCACCACC |
| ***ADIPOQ*** | GGGCCCCAGGCCGTGATGGCA | TCG GGG ACCTTCAGCCCCGGGTA |
| ***LPL*** | CTTGGAGATGTGGACCAGC | GTGCCATACAGAGAAATCTC |
| ***RUNX2*** | TGGTTACTGTCATGGCGGGTA | TCTCAGATCGTTGAACCTTGCTA |
| ***ALPL*** | ACGTGGCTAAGAATGTCATC | CTGGTAGGCGATGTCCTTA |
| ***BGLAP*** | CATGAGAGCCCTCACA | AGAGCGACACCCTAGAC |
| ***IL1B*** | CTCGCCAGTGAAATGATGGCT | GTCGGAGATTCGTAGCTGGAT |
| ***TNFA*** | ATGAGCACTGAAAGCATGATCC | GAGGGCTGATTAGAGAGAGGTC |
| ***NFκB (RELA)*** | GAACCAGGGCATACCTGTGG | TAGCCTCAGGGTACTCCATCA |
| ***TGFB1*** | CTAATGGTGGAAACCCACAACG | TATCGCCAGGAATTGTTGCTG |
| ***TPB3*** | CCTGAGGTTGGCTCTGACTGTA | TGTTCCGTCCCAGTAGATTACCA |
| ***CDKN2A*** | CAGAAATGATCGGAAACCATT | CTACGCATGCCTGCTTCTAC |
| ***VEGFA*** | CCCACTGAGGAGTCCAACATC | GGCCTTGGTGAGGTTTGATC |
| ***IFNG*** | AAACGAGATGACTTCGAAAAGC | ATATTGCAGGCAGGACAACC |
| ***IL6*** | ACTCACCTCTTCAGAACGAATTG | CCATCTTTGGAAGGTTCAGGTTG |
| ***IL10*** | ATGCCCCAAGCTGAGAACCAAGAC | TCTCAAGGGGCTGGGTCAGCTATC |
| ***MMP2*** | AGATCTTCTTCTTCAAGGACCGGTT | GGCTGGTCAGTGGCTTGGGGTA |
| ***MMP9*** | GCGGAGATTGGGAACCAGCTGTA | GACGCGCCTGTGTACACCCACA |
